# Prognostic value of the combined effect of nutritional status and body water component in patients with colorectal cancer

**DOI:** 10.1038/s41598-023-43736-0

**Published:** 2023-10-16

**Authors:** Yining Liu, Xiangliang Liu, Linnan Duan, Yixin Zhao, Yuwei He, Wei Li, Jiuwei Cui

**Affiliations:** 1 Center of Cancer, The First Hospital of Jilin University, Changchun, 130021 China; 2 Department of Neurosurgery, The First Hospital of Jilin University, Changchun, 130021 China

**Keywords:** Cancer, Health care, Oncology

## Abstract

The aim of this study was to explore the impact of Geriatric Nutritional Risk Index (GNRI) and body water component (BWC) on the survival of colorectal cancer (CRC) patients and whether the combined effect had a potential prognostic and predictive efficacy. We evaluated the accuracy of GNRI for malnutrition and estimated the predictive capacity of BWC for survival. Kaplan–Meier survival curves and cox regression analyses were used to examine the prognostic effects. A nutrition-water score (NWS) model was developed and evaluated the survival predictive power. GNRI and extracellular water-to-intracellular water ratio (ECW/ICW) were integrated, with the cut-off values of 103.5 and 63.7%. Lower GNRI and higher ECW/ICW were independent risk factors for poor prognosis in CRC patients. The combination of the two into the NWS model demonstrated a higher risk of death for patients with NWS ≥ 1 compared to those with NWS of 0. NWS showed a better predictive capability compared to GNRI and ECW/ICW, with the concordance index of 0.681. Our study demonstrates GNRI and ECW/ICW’s prognostic utility in CRC, with their combination improving survival prediction to help guide patient-centered treatment.

## Introduction

Colorectal cancer (CRC) ranks as the third most common cancer and cause of cancer mortality worldwide, accounting for approximately 10% of cancer cases and deaths^1^. Despite current advances in the diagnosis and treatment of patients with CRC, simple and reliable prognostic biomarkers for CRC remain lacking. Malnutrition prevails in 30–80% of cancer patients depending on factors like site and stage, severely impacting treatment tolerance, quality of life, and promoting sarcopenia and cachexia^2–5^. Whereas malnutrition is often overlooked by physicians and patients in clinical practice, nutritional assessment tools are necessary for evaluating the nutritional status. Patient-Generated Subjective Global Assessment (PG-SGA) has been shown to be a valid and rapid method widely recognized worldwide, and has been confirmed as a predictive and prognostic factor for short-term and long-term survival of tumor patients^6,7^. Nevertheless, it is complicated and depends to a large extent on the subjective opinions of patients, thus optimized regimens are now developed through objective and accessible serological indicators to assess nutritional conditions. Geriatric Nutritional Risk Index (GNRI) is promising in predicting prognosis and postoperative complications in patients with rectal cancer^8^, and is considered to be an independent factor for sarcopenia in patients with metastatic CRC^9^. Such nutritional markers like GNRI are based on laboratory tests and anthropometric measurements, which have potentials to prognosticate in CRC.

Body water component (BWC) derived from bioelectrical impedance analysis (BIA) provides insight into fluid balance and cell health^10^. BWC alterations like expanded extracellular (ECW) and depleted intracellular water (ICW) associate with disease states^11–14^. ICW is an important determinant of cell volume, mainly present in muscular tissues, and is of great importance in characterizing changes in muscle mass. Patients after dialysis have shown a decrease in ICW with no significant changes in ECW, and low ICW is an independent risk factor for poor prognosis^15^. The ECW-to-ICW ratio (ECW/ICW) combines both ECW excess and ICW loss, increasing with inflammation, malnutrition, and age^13,15,16^. Tumor patients are prone to fluid imbalance and water transfers from inside to outside of the cells due to disease burden and malnutrition, thus monitoring changes in BWC can be useful in keeping track of the health status of tumor patients.

To our knowledge, no studies have examined prognostic value of ECW/ICW in CRC. We therefore aimed to 1) evaluate nutritional indicators and BWC ratios for prognostic utility in CRC and 2) develop an integrative nutrition-BWC scoring system for survival prediction and guiding interventions.

## Methods

### Study populations

The retrospective study enrolled patients with CRC from the First Hospital of Jilin University from January 2013 to December 2019. Inclusion criteria included people aged ≥ 18 years, with a first admission or pathological diagnosis of CRC, Eastern Cooperative Oncology Group (ECOG) score of 0–2, and no prior nutritional support or treatment. Exclusion criteria were set for the study, which included people with a combination of other types of tumors, incomplete clinical records of necessary indexes, death within 30 days of admission, a combination of severe pleural effusion, peritoneal effusion, and those who had received hemodialysis. Patients were followed up regularly by telephone or outpatient visits. The primary endpoint was overall survival (OS), defined as the time from first admission or diagnosis to death or last follow-up.

## Data collection

The baseline data were collected from the electronic medical record system, including general information (age, sex, height, body weight (BW), smoking and alcohol history, family history, comorbidities), medical information (TNM stage, treatments, nutritional support), serological tests (neutrophils, lymphocytes, albumin), PG-SGA, and body composition indices obtained from BIA. Body mass index (BMI) was calculated by weight/ squared of height, and patients were classified as underweight (< 18.5 kg/m^2^), normal weight (18.5–23.9 kg/m^2^), overweight (24.0–27.9 kg/m^2^), and obese (≥ 28 kg/m^2^)^17^. TNM staging was based on the 8th American Joint Committee on Cancer (AJCC) on TNM classification system, and treatments include surgery, chemoradiotherapy, immunotherapy, and targeted therapy. Neutrophil-to-lymphocyte ratio (NLR) was defined by the ratio of neutrophils to lymphocytes.

BIA was performed within 24 h of admission, and all participants wore light clothing and avoided physical activity before measurements. They were fitted with 8 electrodes, and InbodyS10 (BiospaceCo ®) was used to measure resistance and capacitance directly at 50 kHz and 800 mA. BIA was used to measure body composition, including BWC, fat mass (FM), and fat free mass (FFM). ECW/TBW was calculated by dividing ECW by TBW, and ICW/TBW and ECW/ICW were calculated in the same way.

## Nutritional assessment

PG-SGA score was collected at the time of initial admission for nutritional status assessment, a scale completed by patients and physicians, consisting of seven aspects (body weight, eating conditions, symptoms, activity and physical function, nutrition-related disease status, metabolic status and physical examination). Patients were classified as malnutrition (≥ 4) and no malnutrition (< 4) based on PG-SGA. GNRI is a novel nutritional assessment tool obtained from baseline hematological variables, which is calculated as follows:

$$ {{GNRI}} = 1.489 \times {{ALB}}\left( {{{g}}/{{L}}} \right) + 41.7 \times \left[ {{{current}} \,{{BW}}\, \left( {{{kg}}} \right) \div 22 \times {{height}}\,\left( {{m}} \right)^{2} } \right] $$

$${\text{If}}\;\left[ {{{current}}\, {{BW}}\, \left( {{{kg}}} \right) \div 22 \times {{height}}\,\left( {{m}} \right)^{2} } \right]\; > {{1}},{\text{ it was set to 1}}$$^18^.

### Statistical analysis

The data were analysed by SPSS 25.0 statistical software and R Project for Statistical Computing (version 4.2.1). Kolmogorov–Smirnov test (K-S test) was used to examine the normal distribution. Continuous variables were presented as mean ± standard deviation (SD) for normally distributed data, those not eligible were presented as median (interquartile range, IQR), and categorical variables were presented as count (percentage, %). The accuracy of GNRI for the diagnosis of malnutrition was assessed and statistically described. Time-dependent Receiver Operating Characteristic (ROC) curves and area under the curve (AUC) were used to assess the predictive ability of different water components on survival, and restrictive cubic spline (RCS) were applied to reflect the non-linear relationship between BWC and the risk of death in CRC patients. The cut-off value of ECW/ICW and GNRI were based on standardized log-rank statistics using the “survminer” package, with the optimal thresholds for 63.7% and 103.5, respectively. Associations between baseline variables and ECW/ICW were analysed by multivariate logistic regression. The effects of nutritional and water components on survival were assessed using Kaplan–Meier survival curves and statistically tested using log-rank analysis, and after adjusting for confounders, risk ratios (HRs) and 95% confidence intervals (95% CI) were estimated using cox regression analysis and then subgroup analyses were performed. Following this, we established a nutrition-water score (NWS), and evaluated it with a survival prognostic model, in addition, the concordance index (C-index) was used to measure the discriminative capacity of the scoring system. Finally, sensitivity analyses were conducted to further assess the prognostic value of the model.

### Ethics approval and consent to participate

The study was approved by the Ethics Committee of the First Hospital of Jilin University (2017–362), and all patient data in this study were approved by this committee. All methods were performed in accordance with the relevant guidelines and regulations. And informed consent was obtained from all patients.

## Results

### Patients characteristics

A total of 507 CRC patients were enrolled, of whom 291 (57.4%) were males, and the median age (IQR) was 59.0 (13.0) years. 76.3% of the participants were in TNM stage III and IV, and the numbers receiving surgery, chemotherapy and radiotherapy were 402, 309 and 21, respectively. The incidence of malnutrition based on PG-SGA was 66.9% and when it comes to GNRI, the percentage was 61.9%. Additional baseline information like comorbidities and family history were detailed in Table 1.

**Table 1:** Baseline characteristics of CRC patients.

Characteristics	Overall (*N* = 507)	ECW/ICW		
		low (*N* = 259)	high (*N* = 248)	*P*
Age, y	59.0 (13.0)	56.0 (15.0)	62.0 (12.0)	< 0.001
≥ 65	135 (26.6)	39 (15.1)	96 (38.7)	< 0.001
< 65	372 (73.4)	220 (84.9)	152 (61.3)	
Sex				0.063
Male	291 (57.4)	159 (61.4)	132 (53.2)	
Female	216 (42.6)	100 (38.6)	116 (46.8)	
Smoking (yes), *n* (%)	194 (38.3)	98 (37.8)	96 (38.7)	0.84
Drinking (yes), *n* (%)	101 (19.9)	54 (20.8)	47 (19.0)	0.593
Comorbidities (yes), *n* (%)	204 (40.2)	83 (32.0)	121 (40.2)	< 0.001
Diabetes, *n* (%)	55 (10.8)	23 (8.9)	32 (12.9)	
Hypertension, *n* (%)	102 (20.1)	37 (14.3)	65 (26.2)	
Inflammatory bowel disease (IBD), *n* (%)	16 (3.2)	4 (1.5)	12 (4.8)	
Chronic heart disease (CHD), *n* (%)	22 (4.3)	10 (3.9)	12 (4.8)	
Chronic hepatobiliary disease (CHBD), *n* (%)	37 (7.3)	19 (7.3)	18 (7.3)	
Family history (yes), *n* (%)	103 (20.3)	58 (22.4)	45 (18.1)	0.235
TNM stage				0.01
I	12 (2.4)	9 (3.5)	3 (1.2)	
II	103 (20.3)	61 (23.6)	42 (16.9)	
III	253 (49.9)	132 (51.0)	121 (48.8)	
IV	139 (27.4)	57 (22.0)	82 (33.1)	
Treatment				
Surgery (yes), *n* (%)	402 (79.3)	219 (84.6)	183 (73.8)	0.003
Chemotherapy (yes), *n* (%)	309 (60.9)	153 (59.1)	156 (62.9)	0.377
Radiotherapy (yes), *n* (%)	21 (4.1)	9 (3.5)	12 (4.8)	0.441
Targeted therapy (yes), *n* (%)	42 (8.3)	20 (7.7)	22 (8.9)	0.639
Immunotherapy (yes), *n* (%)	17 (3.4)	9 (3.5)	8 (3.2)	0.876
Nutritional support (yes), *n* (%)	207 (40.8)	106 (40.9)	101 (40.7)	0.963
Enteral nutrition	88 (17.4)	65 (25.1)	67 (27.0)	0.622
Parenteral nutrition	132 (26.0)	44 (17.0)	44 (17.7)	0.823
NRS2002				0.003
≥ 3	400 (78.9)	41 (15.8)	66 (26.6)	
< 3	107 (21.1)	218 (84.2)	182 (73.4)	
BMI, as continuous, kg/m2	22.88 (3.4)	23.58 (3.3)	22.14 (3.3)	< 0.001
Category				< 0.001
< 18.5	46 (9.1)	12 (4.6)	34 (13.7)	
18.5–23.9	280 (55.2)	132 (51.0)	148 (59.7)	
24–27.9	148 (29.2)	92 (35.5)	56 (22.6)	
≥ 28	33 (6.5)	23 (8.9)	10 (4.0)	
FM, kg	15.75 (8.50)	16.5 (9.2)	15.1 (8.7)	0.01
FFM, kg	46.90 (13.52)	49.3 (13.5)	44.3 (12.7)	< 0.001
NLR	2.40 (2.70)	2.35 (2.40)	2.41 (2.92)	0.801
PG-SGA, as continuous	6.00 (6.00)	5.00 (6.00)	6.00 (6.00)	0.001
Category				0.035
Normal(< 4)	168 (33.1)	97 (37.5)	71 (28.6)	
Malnutrition (≥ 4)	339 (66.9)	162 (62.5)	177 (71.4)	
GNRI, as continuous	100.53 (12.92)	103.03 (12.76)	98.15 (13.21)	< 0.001
Category				< 0.001
Normal(≥ 103.5)	193 (38.1)	124 (47.9)	69 (27.8)	
Malnutrition(< 103.5)	314 (61.9)	135 (52.1)	179 (72.2)	
ECW	13.50 (3.70)	13.8 (4.0)	13.0 (3.6)	0.055
ICW	20.95 (6.30)	22.6 (6.3)	19.7 (5.6)	< 0.001
ECW/TBW	0.39 (0.01)	0.38 (0.01)	0.39 (0.01)	< 0.001
ICW/TBW	0.61 (0.01)	0.62 (0.01)	0.61 (0.01)	< 0.001
ECW/ICW	0.64 (0.03)	0.62 (0.02)	0.65 (0.03)	< 0.001

Values are presented as mean (standard deviation, SD), or median (interquartile range, IQR) or n (%). *NRS2002*, Nutritional Risk Screening 2002; *BMI*, body mass index; *FM*, fat mass; *FFM*, fat free mass; *NLR*, neutrophil-to-lymphocyte ratio; *PG-SGA*, Patient-generated Subjective Global Assessment; *GNRI*, geriatric nutritional risk index; *ECW*, extracellular water; *ICW*, intracellular water; *ECW/TBW*, extracellular water to total body water ratio; *ICW/TBW*, intracellular water to total body water ratio; *ECW/ICW*, extracellular water to intracellular water ratio.

## Analysis of nutritional assessment tools

We evaluated the accuracy of the nutritional indicator, GNRI for the diagnosis of malnutrition, with the AUC of 0.718, and the sensitivity and the concordance was listed in Supplementary Table S1. Then we analysed the relationship between nutritional assessment tools, a total of 244 patients could be diagnosed as malnourished by both PG-SGA and GNRI, while 98 patients were not defined as malnutrition by any of the nutritional assessment tools.

## Selection and comparison of BWC

**Figure 1:**
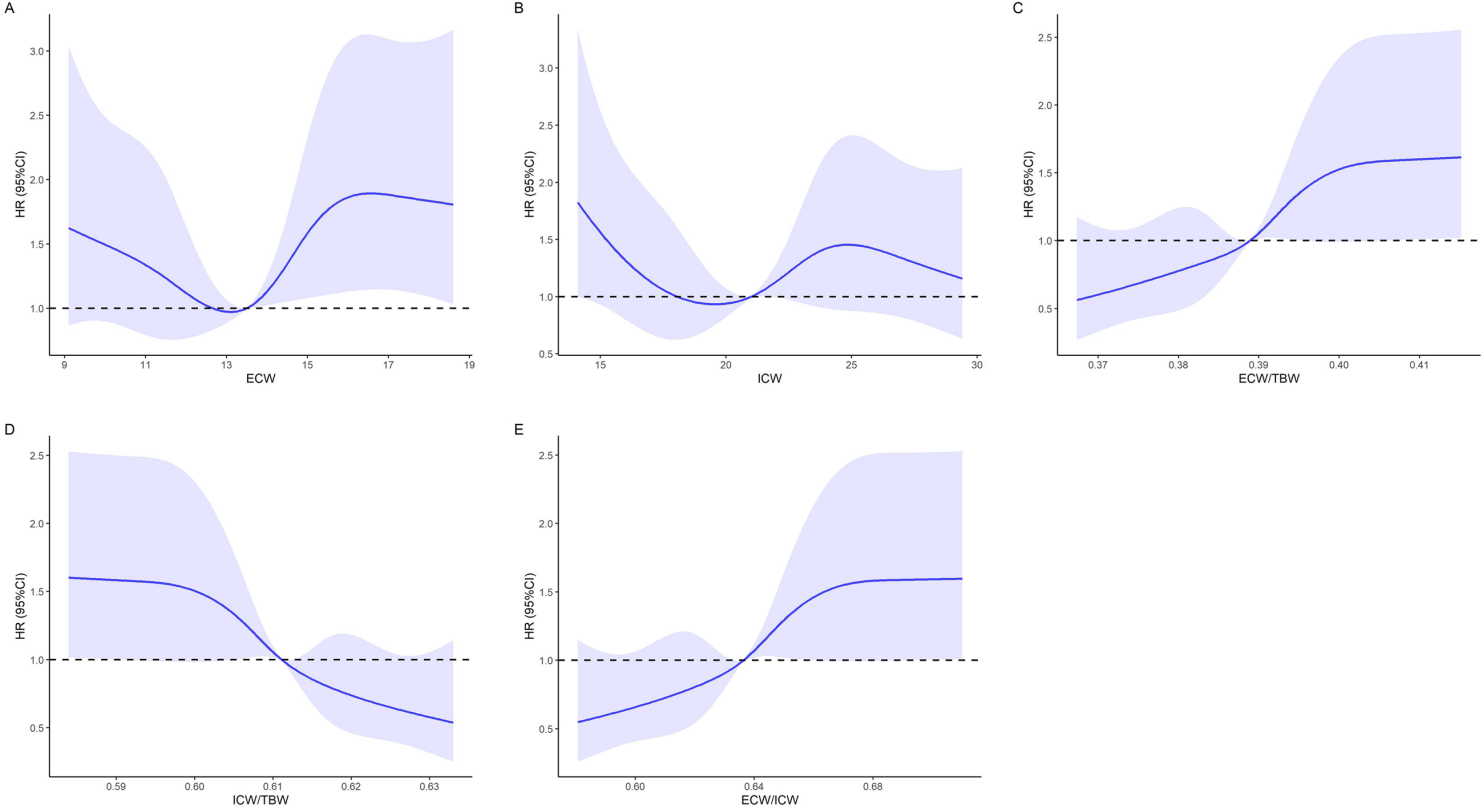
The association between BWC and mortality of CRC. (**A**) ECW, extracellular water; (**B**) ICW, intracellular water; (**C**) ECW/TBW, extracellular water to total body water ratio; (**D**) ICW/TBW, intracellular water to total body water ratio; (**E**) ECW/ICW, extracellular water to intracellular water ratio. BWC, body water composition; CRC, colorectal cancer.

Several body water-related variables were chosen to characterised the predictive capability for survival using time-dependent ROC curves (see Supplementary Table S2 for details), with ECW/TBW and ECW/ICW having the highest AUCs of 0.634 (0.573–0.695). RCS revealed a non-linear relationship between ECW/TBW, ICW/TBW and ECW/ICW and all-cause mortality in CRC patients (Fig. 1). Combined with the nutritional and metabolic characteristics of tumor patients, we further investigated the role of ECW/ICW. Patients was divided into high ECW/ICW group (E/I group) and low E/I group on the basis of the cut-off value, as demonstrated in Table 1 and Supplementary Fig. S1. Age, comorbidities, TNM stage, surgery, Nutritional Risk Screening 2002 (NRS2002), BMI, FM, FFM and GNRI were significantly different between the two groups, and the above variables were included in a multivariate logistic regression. The results showed that elderly age, comorbidities, higher nutrition risks were associated with higher ECW/ICW, while FFM and GNRI were protective factors in keeping fluid balance in the body (OR = 0.975, 95% CI, [0.951–1.000]; OR = 0.961, 95% CI, [0.934–0.990]).

## Association between GNRI and ECW/ICW and survival

**Figure 2:**
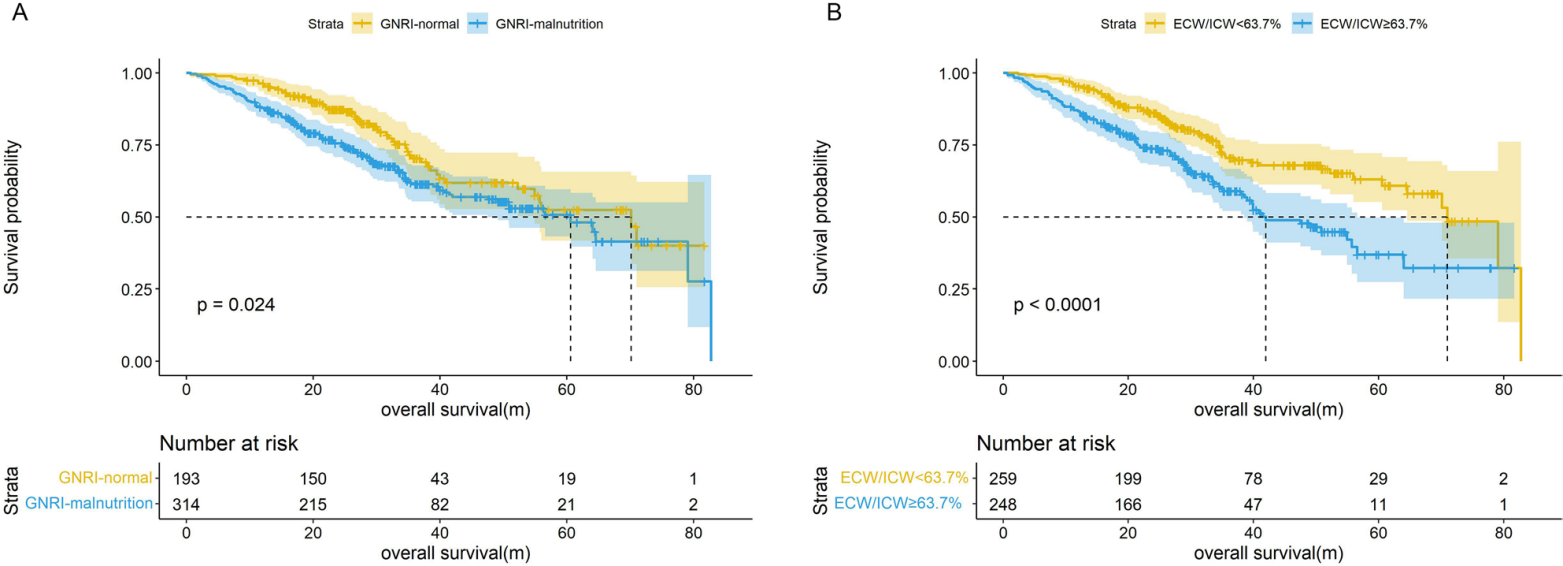
Cox regression analysis of GNRI and ECW/ICW associated with overall survival. (**A**) GNRI, geriatric nutritional risk index; (**B**) ECW/ICW, extracellular water to intracellular water ratio.

Patients were separated into malnourished (< 103.5) and well-nourished (≥ 103.5) groups by GNRI. The Kaplan–Meier curve showed that GNRI-defined malnutrition was associated with a shorter survival time (*p* = 0.024), and by incorporating GNRI and other confounding factors into a multivariate cox regression analysis, the results indicated that low GNRI was an independent risk factor for poor prognosis (HR = 1.762, 95% CI, [1.141–2.720]). Furthermore, in a subgroup of females, age over 65 years, TNM stage in III-IV, surgery, chemoradiotherapy, PG-SGA-diagnosed malnutrition, obesity or overweight, low GNRI was independently associated with an increased risk of death compared to a high GNRI. We subsequently performed an analysis of ECW/ICW on survival in CRC patients, which revealed that ECW/ICW ≥ 63.7% was an independent predictor of worse prognosis (HR = 1.477, 95% CI, [1.030–2.118]), and stratified analyses similarly showed an association between ECW/ICW and mortality in CRC patients, as detailed in Fig. 2, Table 2 and Supplementary Fig. S2.

**Table 2:** Cox regression analysis of GNRI and ECW/ICW associated with overall survival.

	Crude		Model A		Model B		Model C	
	HR (95%CI)	*P*	HR (95%CI)	*P*	HR (95%CI)	*P*	HR (95%CI)	*P*
*GNRI*								
≥ 103.5	Ref		Ref		Ref		Ref	
< 103.5	1.455 (1.049–2.018)	0.025	1.441 (1.033–2.010)	0.032	1.448 (1.023–2.050)	0.037	1.762 (1.141–2.720)	0.011
*ECW/ICW*								
< 63.7%	Ref		Ref		Ref		Ref	
≥ 63.7%	1.859 (1.366–2.528)	< 0.001	1.895 (1.366–2.629)	< 0.001	1.533 (1.093–2.150)	0.013	1.477 (1.030–2.118)	0.034

Model A: adjusted for age and sex.

Model B: adjusted for model A plus TNM stage, comorbidities, smoking and drinking history, family history, surgery, chemotherapy, radiotherapy, targeted therapy, immunotherapy and nutritional support.

Model C: adjusted for model B plus PG-SGA, NRS2002, BMI, NLR, FM and FFM, with GNRI and ECW/ICW for mutual adjustment.

## Prognostic and predictive value of NWS in CRC

**Figure 3:**
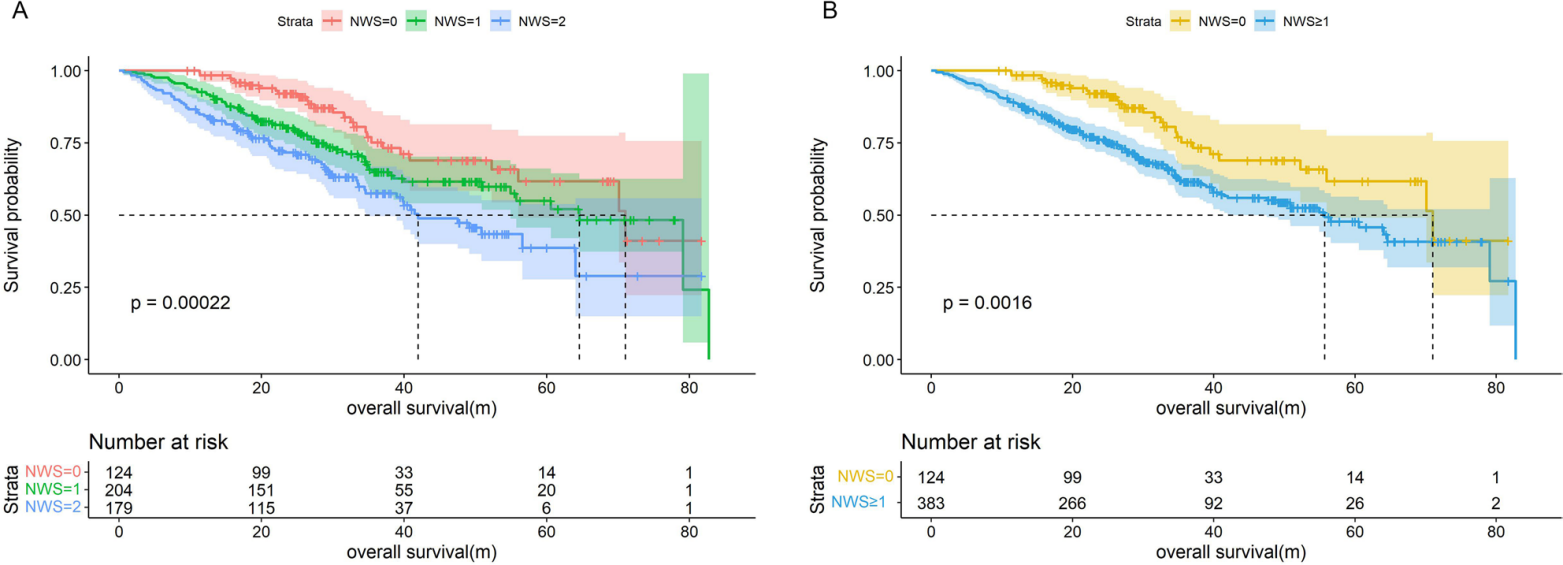
Kaplan–Meier curves for overall survival of Nutrition-Water score. (**A**) Categorized by NWS = 0,1,2; (**B**) Categorized by NWS = 0,1.

**Figure 4:**
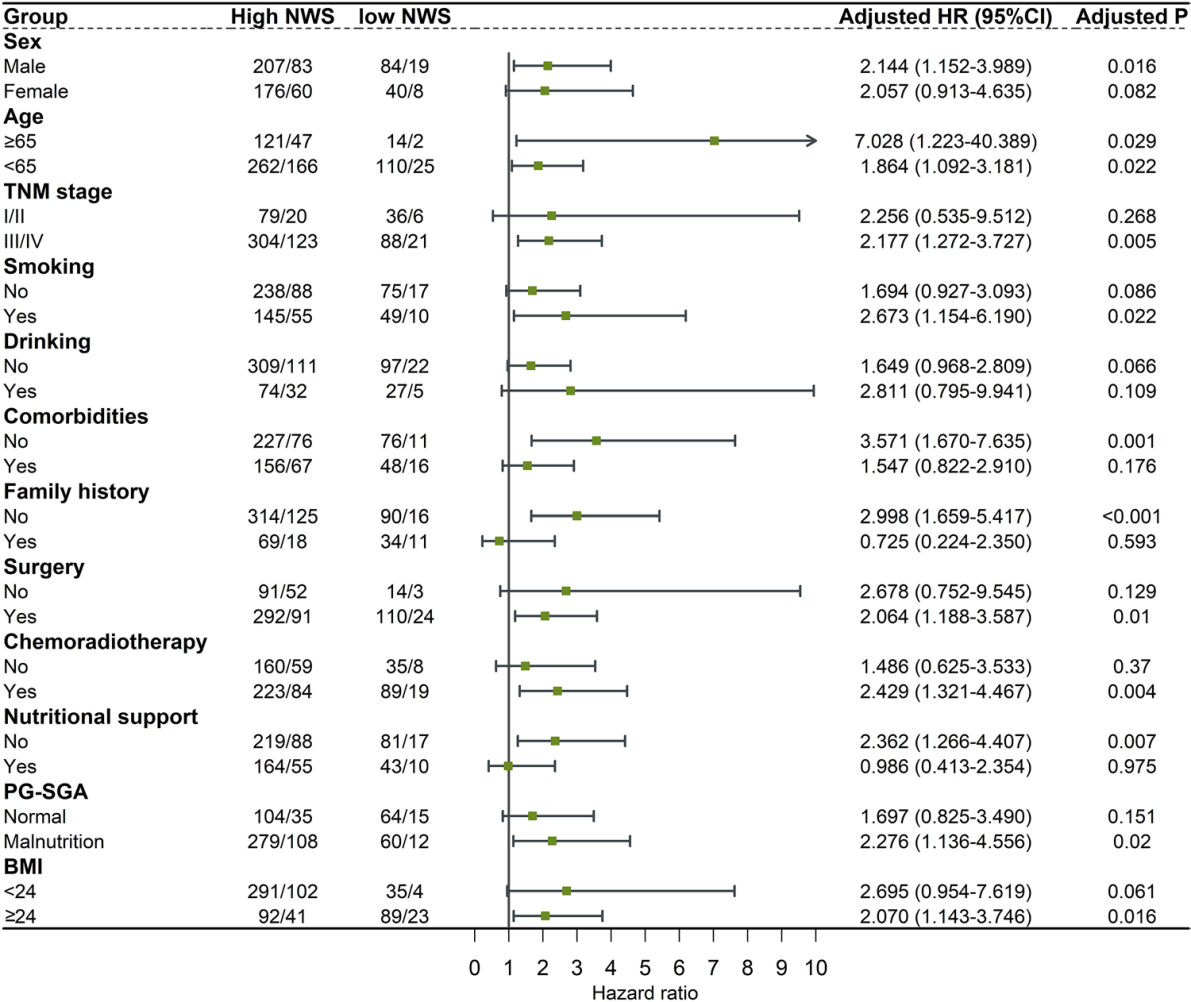
Subgroups analyses of the association between Nutrition-Water score and hazard risk of overall survival after adjusted for confounding factors. Each subgroup analysis adjusted for age, sex, TNM stage, comorbidities, smoking and drinking history, family history, treatment, nutritional support, PG-SGA, NRS2002, BMI, NLR, FM and FFM. PG-SGA, Patient-generated Subjective Global Assessment; NRS2002, Nutritional Risk Screening 2002; BMI, body mass index; FM, fat mass; FFM, fat free mass.

We developed a NWS with two variables, GNRI and ECW/ICW, as shown in Table 3, where NWS was 0 for patients with GNRI ≥ 103.5 and ECW/ICW < 63.7%, 2 for patients with GNRI < 103.5 and ECW/ICW ≥ 63.7%, and NWS was defined as 1 for others. As compared to patients with NWS of 0, patients with NWS of 1 and 2 had an elevated mortality risk, with HRs of 1.877 and 2.636, respectively, and for patients with NWS ≥ 1, HR (95% CI) of all-cause mortality was 2.021 (1.248–3.274). Kaplan–Meier curves showed that patients with NWS of 0 had longer overall survival and similar results were obtained in subgroup analyses, such as age, sex and TNM stage (Fig. 3, Fig. 4, Table 4 and Supplementary Fig. S3). Supplementary Table S3 illustrated the predictive ability of survival for GNRI, ECW/ICW and NWS, with lower C-indexes for GNRI (0.622) and ECW/ICW (0.634) than for NWS (0.681). Sensitivity analyses showed that after excluding CRC patients who died within 3 and 6 months, NWS ≥ 1 remained a promising predictor of survival, which was associated with a relatively high hazard of death (Supplementary Table S4).

**Table 3:** Development of Nutrition-Water score (NWS).

	ECW/ICW	GNRI	No
*NWS*			
0	< 63.7%	≥ 103.5	124
1	ECW/ICW ≥ 63.7% or GNRI < 103.5	204	
2	≥ 63.7%	< 103.5	179

GNRI, geriatric nutritional risk index; ECW/ICW, extracellular water to intracellular water ratio.

**Table 4:** Cox regression analysis of Nutrition-Water score (NWS) and overall survival.

	Crude		Model A		Model B		Model C	
	HR (95%CI)	P	HR (95%CI)	P	HR (95%CI)	P	HR (95%CI)	P
*NWS*								
0	Ref		Ref		Ref		Ref	
1	1.584 (1.014–2.474)	0.043	1.636 (1.038–2.579)	0.034	1.555 (0.983–2.462)	0.059	1.877 (1.143–3.081)	0.013
2	2.389 (1.535–3.717)	< 0.001	2.473 (1.552–3.941)	< 0.001	2.021 (1.259–3.243)	0.004	2.636 (1.505–4.618)	0.001
P for trend		< 0.001		< 0.001		0.013		0.003
*NWS*								
0	Ref		Ref		Ref		Ref	
≥ 1	1.917 (1.270–2.895)	0.002	1.940 (1.267–2.970)	0.002	1.735 (1.127–2.671)	0.012	2.021 (1.248–3.274)	0.004

Model A: adjusted for age and sex.

Model B: adjusted for model A plus TNM stage, comorbidities, smoking and drinking history, family history, surgery, chemotherapy, radiotherapy, targeted therapy, immunotherapy and nutritional support.

Model C: adjusted for model B plus PG-SGA, NRS2002, BMI, NLR, FM and FFM.

## Discussion

To our knowledge, this is the first study to explore the combined effect of nutritional status and water components on the survival of CRC patients. We found that low GNRI and high ECW/ICW were independent prognostic factors for poor survival in CRC patients, and after developing a NWS model based on the two, it showed that NWS was negatively correlated with prognosis and more predictive of survival than the two variables alone.

Our findings confirm nutritional screening's importance in cancer, with PG-SGA and GNRI both predicting survival like prior studies^19,20^. GNRI offers a convenient serum index, correlating closely with PG-SGA as it incorporates ALB and weight^8,9,21^. However, a major weakness of ALB as a nutritional marker is its long half-life of about 20 days and is affected by inflammation and fluid balance^22^. Under acute stresses such as infection and injury, the catabolism of ALB is increased and serum ALB substantially infiltrates into the interstitial space; therefore, there is no significant correlation between ALB and nutritional status in the acute phase^23^. GNRI-defined malnutrition associated with higher mortality, likely reflecting impaired immunity from hypoalbuminemia and inflammation from cytokines like IL-1, IL-6, and TNF-α^24,25^. We observed low GNRI conferring higher mortality with surgery and chemoradiotherapy, highlighting malnutrition's detrimental interactions with therapy through worsened side effects and recovery^4,26,27^. Malnutrition prolongs hospital stay and reduces treatment tolerance, while chemotherapy has an increased nutritional risk, that kills tumor cells but simultaneously induces gastrointestinal adverse reactions such as vomiting and diarrhoea, exacerbating nutritional depletion^28^. Likewise, as surgery is the first choice for CRC patients, postoperative dysfunction of the gastrointestinal tract results in a reduced anabolic response, impairing nutrient absorption such as amino acids and protein synthesis within muscle tissue^14^. Early identification and management of malnutrition are increasingly recognized as integral in cancer care.

Studies have suggested that BWC is closely related to the health status of the body^29,30^, and the input and output of cellular water are kept in dynamic balance to maintain the internal environment in homeostasis^31^. Our study provides initial evidence that higher ECW/ICW independently associates with worse CRC survival. This likely results from apoptosis decreasing cell volume and shifting fluids^12^. In cancer, reduced intake, cachexia, and inflammation disrupt cell membranes, elevating permeability and accelerating ICW loss and edema^11,31^. Depleted ICW impairs metabolism and protein function while promoting muscle catabolism^30,32^. Simultaneously, ECW excess indicates underlying pathology. It has been shown that ECW/ICW is highly related to malnutrition and inflammatory response and increased with age^13,16^. Furthermore, we observed gender differences in this risk factor, with the correlation between ECW/ICW and prognosis more likely to occur in females, which could be explained by sex hormone differences due to different expression levels of estrogen and androgen receptors, and that females in dehydration are more prone to tumor progression^33^. Consequently, early BWC assessment may thus enhance prognostication.

Cancer patients, especially the elderly, are susceptible to worsening nutritional status during therapy^4^. On the one hand, malnutrition causes ICW and muscle loss, while cachexia accelerates proteolysis and water loss^31^. Hypoalbuminemia further promotes fluid shifts. On the other hand, a decrease in skeletal muscle mass and function is an important hallmark of sarcopenia and is related to a higher risk of death in tumor patients^27^. Muscle loss leads to a decrease in ICW, which further affects synthesis of nutrients in the cells and perpetuates malnutrition^32^. Moreover, tumor-related inflammation leads to intracellular fluid transfer to the extracellular compartment via increased cell membrane permeability, and impedes nutrient uptake^34^. However, relationships between nutrition and water metrics remain unexplored. Our integrated NWS model combining GNRI and ECW/ICW showed enhanced predictive power over either alone, with higher NWS independently predicting poorer survival. This effect was greater in older males and later stages, highlighting interrelationships between nutrition, water, age, sex, and stage^34^, and that the volume of the body water compartment may vary evidently from patient to another. Elderly patients' reduced energy needs and treatment effects promote malnutrition and water-muscle loss^19^. Sarcopenia research reveals progressive muscle decline with age related to weakness and frailty^35^. Higher muscle ICW associates with better function and lower frailty in elderly survivors^32^. Patients with advanced tumors are in a state of high nutritional exhaustion and cachexia, with an imbalance in fluid distribution, leading to shorter survival times and reduced quality of life. Incorporating both nutrition and water metrics may provide valuable prognostic data to guide care, warranting research in other cancers. We recommend nutritional support for high ECW/ICW to improve cellular volume tolerance, which has been confirmed by other studies^36,37^.

Limitations include the single-center retrospective design, sample size, potential biases, and lack of inflammation data. Additional longitudinal and external validation is needed. Nevertheless, our study confirms the independent impact of GNRI and ECW/ICW on survival in CRC patients, and their combination owns more potential predictive values. In the era of precision medicine, simple and easy-to-use nutritional assessment methods and BWC provide more information on prognosis and help tailor individualized treatment and follow-up strategies, which may be applicable to other tumor types.

### Supplementary Information

Supplementary Information.

## Data Availability

The datasets used and/or analysed during the current study are available from the corresponding author on reasonable request.
