# Supplementary material for: Prognostic value of the combined effect of nutritional status and body water component in patients with colorectal cancer
Source: Sci Rep. 2023 Oct 16;13:17570. doi: 10.1038/s41598-023-43736-0 (PMC10579348; doi:10.1038/s41598-023-43736-0)

Supplementary Information file

Prognostic value of the combined effect of nutritional status and

body water component in patients with colorectal cancer

Authors’ information in order

Yining Liu^1^, Xiangliang Liu^1^, Linnan Duan^2^, Yixin Zhao^1^, Yuwei He^1^, Wei Li^1^*, Jiuwei

Cui^1^*

1

Center of Cancer, The First Hospital of Jilin University, Changchun 130021,

China; [liuyining0820@163.com](mailto:liuyining0820@163.com) (Yining Liu); [ds9291@qq.com](mailto:ds9291@qq.com) (Xiangliang Liu);

[913941214@qq.com](mailto:913941214@qq.com) (Yixin Zhao); [1468335124@qq.com](mailto:1468335124@qq.com) (Yuwei He)

2

Department of Neurosurgery, The First Hospital of Jilin University, Changchun 130021,

China; [duanln1997@163.com](mailto:duanln1997@163.com) (Linnan Duan)

Yining Liu and Xiangliang Liu have contributed equally to this work.

Correspondence information

Correspondence author 1#: Wei Li

Address: Jilin University, Xinmin St No 71, 130021 Changchun, China

Telephone Number: +86137566612

Fax Number: 0431-85619254

E-mail Address: [liwei66@jlu.edu.cn](mailto:liwei66@jlu.edu.cn)

Correspondence author 2#: Jiuwei Cui

Address: Jilin University, Xinmin St No 71, 130021 Changchun, China

Telephone Number: +8613844095420

Fax Number: 0431-85619254

E-mail Address: [cuijw@jlu.edu.cn](mailto:cuijw@jlu.edu.cn)


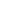


Supplementary Figures

Supplementary Fig. S1 Multivariate logistic regression analysis of the

relevance of baseline variables to ECW/ICW. NRS2002, Nutritional Risk

Screening 2002; BMI, body mass index; FM, fat mass; FFM, fat free mass; PG-SGA,

Patient-generated Subjective Global Assessment; GNRI, geriatric nutritional risk

index; ECW/ICW, extracellular water to intracellular water ratio.


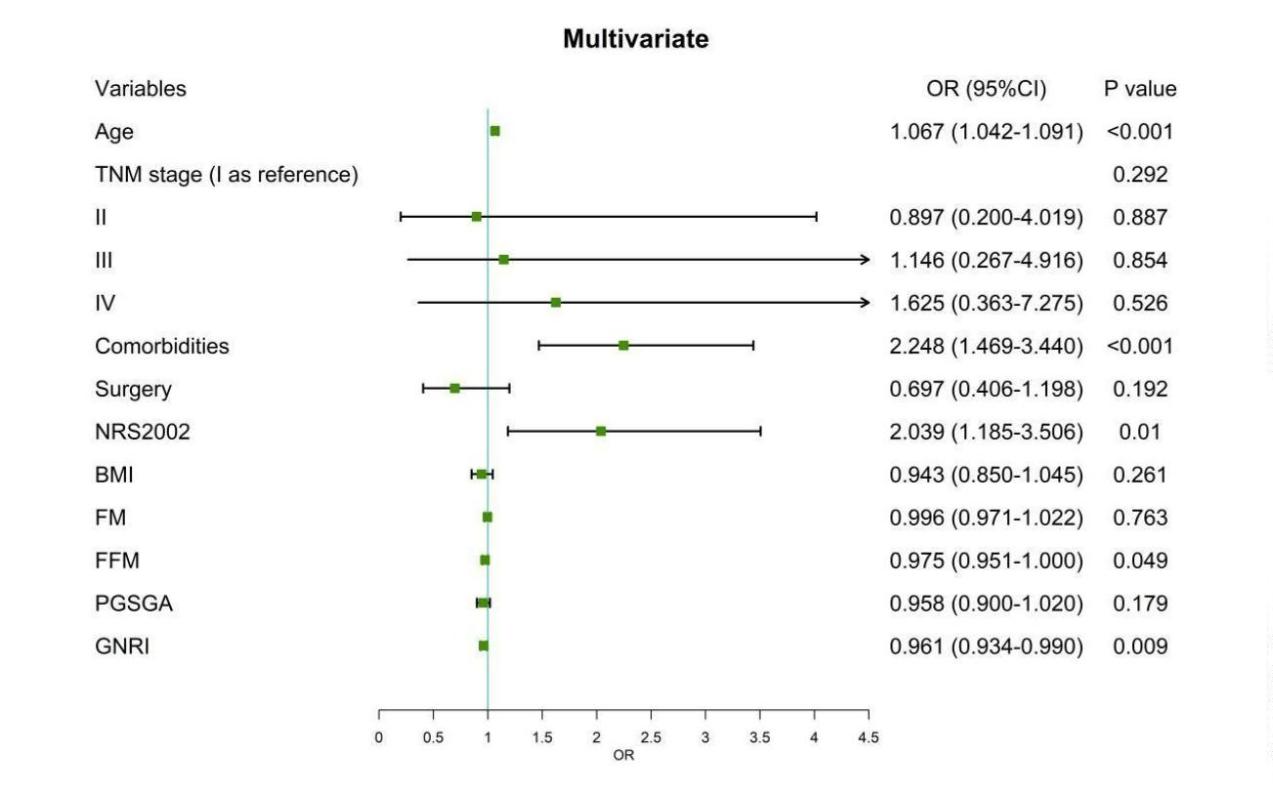

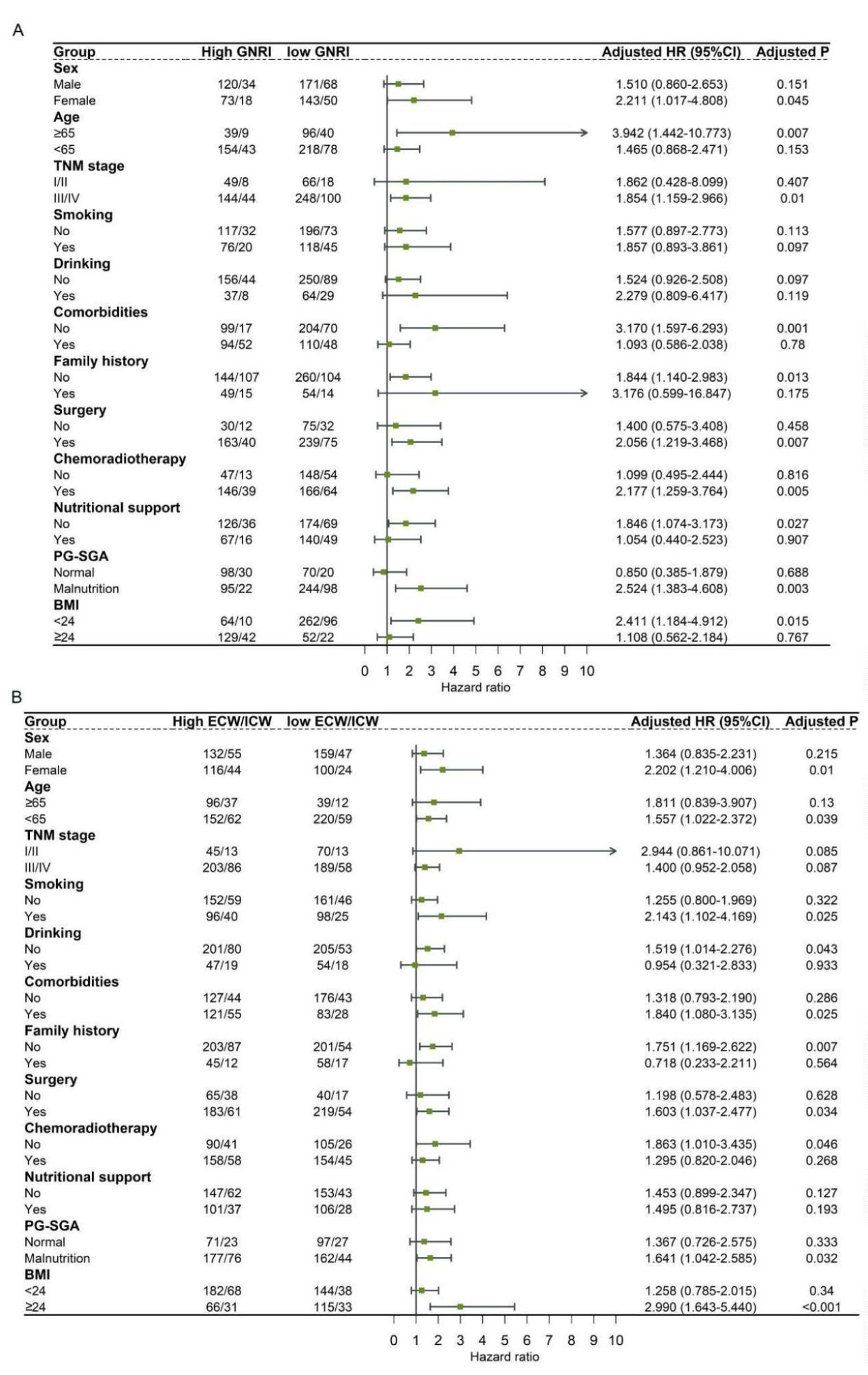


Supplementary Fig. S2 Subgroups analyses of the association between GNRI,

ECW/ICW and OS after adjusted for confounding factors. GNRI, geriatric

nutritional risk index; ECW/ICW, extracellular water to intracellular water ratio; OS,

overall survival; PG-SGA, Patient-generated Subjective Global Assessment; BMI,

body mass index.

Supplementary Fig. S3 Kaplan-Meier curves for overall survival of

Nutrition-Water score in various groups.


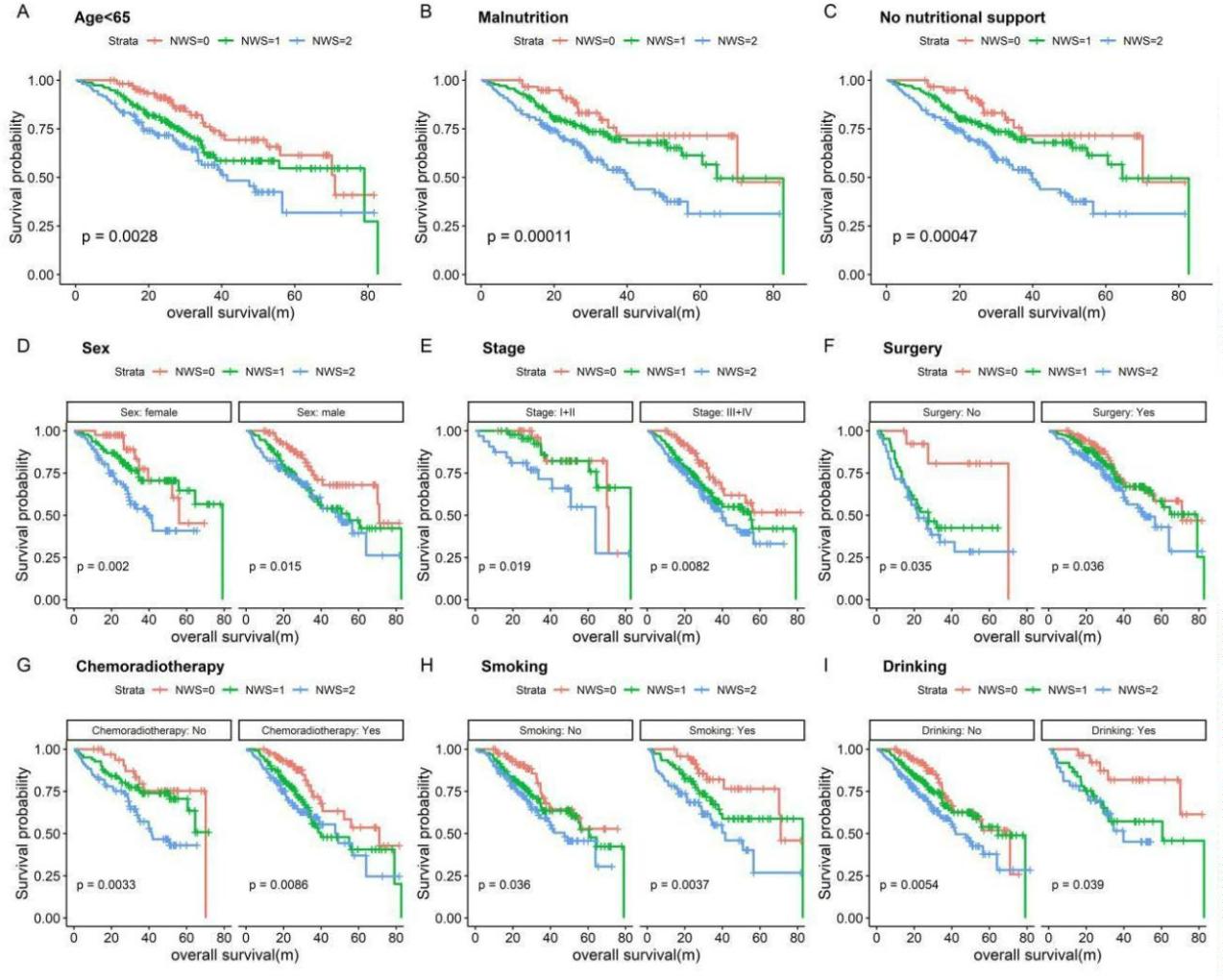


Supplementary Tables

Supplementary Table S1 Statistical descriptions of GNRI for the diagnosis of

malnutrition based on PG-SGA.

GNRI

AUC (95%CI)

Sensitivity

0.718 (0.673-0.763)

0.720

Specificity

0.583

Positive predictive value

Negative predictive value

Kappa (95%CI)

P

0.778

0.508

0.292 (0.206-0.378)

<0.001

GNRI, geriatric nutritional risk index; PG-SGA, Patient-generated Subjective Global Assessment

Supplementary Table S2 AUCs and 95% CI of different BWC

AUC

0.532

0.497

0.634

0.366

0.634

95%CI

ECW

(0.463-0.602)

(0.428-0.565)

(0.573-0.695)

(0.305-0.427)

(0.573-0.695)

ICW

ECW/TBW

ICW/TBW

ECW/ICW

ECW, extracellular water; ICW, intracellular water; ECW/TBW, extracellular water to total body

water ratio; ICW/TBW, intracellular water to total body water ratio; ECW/ICW, extracellular

water to intracellular water ratio.

Supplementary Table S3 Predictive value of Nutrition-Water score for mortality in

patients with colorectal cancer

C-index

0.622

95%CI

GNRI

(0.565-0.680)

(0.573-0.695)

(0.615-0.747)

ECW/ICW

NWS

0.634

0.681


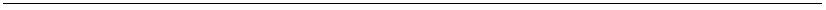

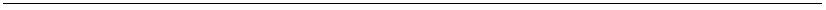

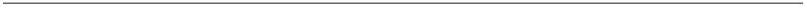

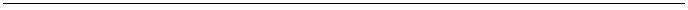

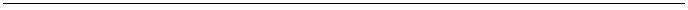

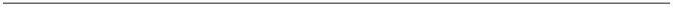

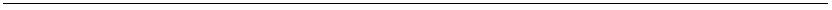

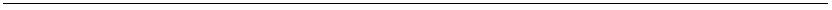

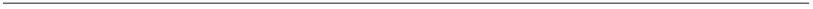


GNRI, geriatric nutritional risk index; ECW/ICW, extracellular water to intracellular water ratio;

NWS, nutrition-water score.

Supplementary Table S4 Sensitivity analyses

Supplementary Table S4a Removal of deaths within 3 months

Crude

Model A

Model B

Model C

HR (95%CI)

P

HR (95%CI)

P

HR (95%CI)

P

HR (95%CI)

P

NWS

0

1

Ref.

Ref.

Ref.

Ref.

1.538

1.607

1.538

1.909

0

.059

0.042

0.067

0.011

(0.983-2.408)

(1.017-2.539)

2.401

(0.970-2.440)

1.976

(1.158-3.147)

2.715

2

2

.277

<

0.001

0.001

<0.001

0.001

0.005

0.019

0.001

0.003

(1.458-3.555)

(1.500-3.842)

(1.226-3.184)

(1.535-4.799)

P for trend

<

NWS

0

Ref.

Ref.

Ref.

Ref.

≥

1

1

.843

1.893

1.706

2.054

0

.004

0.003

0.016

0.004

(1.219-2.787)

(1.234-2.904)

(1.106-2.632)

(1.262-3.342)

Supplementary Table S4b Removal of deaths within 6 months

Crude

Model A

Model B

Model C

HR (95%CI)

P

HR (95%CI)

P

HR (95%CI)

P

HR (95%CI)

P

NWS

0

1

Ref.

Ref.

Ref.

Ref.

1.470

1.522

1.476

1.795

0.094

0.074

0.1

0.023

(0.936-2.308)

(0.960-2.415)

(0.928-2.328)

(1.083-2.975)


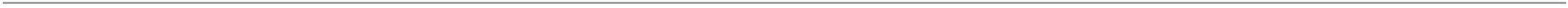

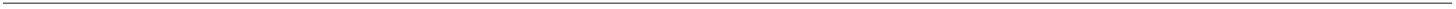

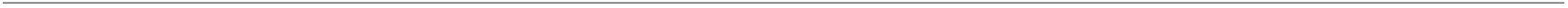

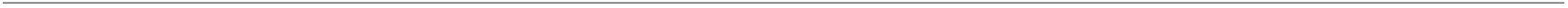

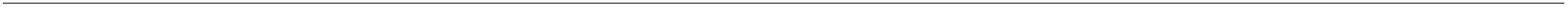

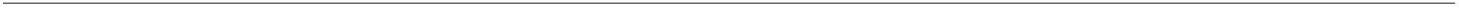

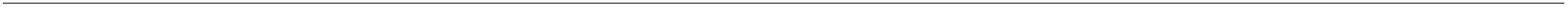


2

2.043

2.157

1.797

2.417

0

.002

0.002

0.006

0.018

0.061

0.003

0.013

(1.298-3.215)

(1.335-3.485)

(1.105-2.921)

(1.347-4.337)

P for trend

0.007

NWS

0

Ref.

Ref.

Ref.

Ref.

≥1

1.705

1.748

1.598

1.903

0.012

0.011

0.035

0.011

(1.124-2.586)

(1.135-2.693)

(1.033-2.473)

(1.162-3.118)


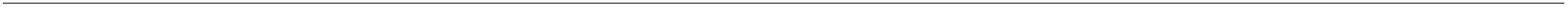

Supplement: Supplementary file 1 — Supplementary Information. [file 41598_2023_43736_MOESM1_ESM.docx]
